# Phase 1 study of new formulation of patritumab (U3-1287) Process 2, a fully human anti-HER3 monoclonal antibody in combination with erlotinib in Japanese patients with advanced non-small cell lung cancer

**DOI:** 10.1007/s00280-016-3231-3

**Published:** 2017-01-31

**Authors:** Toshio Shimizu, Kimio Yonesaka, Hidetoshi Hayashi, Tsutomu Iwasa, Koji Haratani, Hironori Yamada, Shoichi Ohwada, Emi Kamiyama, Kazuhiko Nakagawa

**Affiliations:** 1 Department of Medical Oncology, Kindai University Faculty of Medicine, 377-2, Ohno-higashi, Osaka-sayama, Osaka 589-8511 Japan; 2 Department of Experimental Therapeutics, National Cancer Center Hospital, 5-1-1, Tsukiji, Chuo-ku, Tokyo, 104-0045 Japan; 3 Oncology Clinical Development Department, Daiichi Sankyo Co., Ltd., 1-2-58, Hiromachi, Shinagawa-ku, Tokyo, 140-8710 Japan; 4 Biostatistics and Data Management Department, Daiichi Sankyo Co., Ltd., 1-2-58, Hiromachi, Shinagawa-ku, Tokyo, 140-8710 Japan; 5 Translational Medicine and Clinical Pharmacology Department, Daiichi Sankyo Co., Ltd., 1-2-58, Hiromachi, Shinagawa-ku, Tokyo, 140-8710 Japan

**Keywords:** Patritumab, Anti-HER3 monoclonal antibody, Pharmacokinetics, Phase 1 study

## Abstract

**Background:**

This phase 1 study evaluated the safety, tolerability, pharmacokinetics and efficacy of patritumab (U3-1287) Process 2, a new formulation of fully human anti-HER3 monoclonal antibody in combination with erlotinib, an epidermal growth factor receptortyrosine kinase inhibitor (EGFR-TKI) in prior chemotherapy treated Japanese patients with advanced non-small cell lung cancer (NSCLC).

**Methods:**

Patients received intravenous patritumab Process 2 formulation at 9 mg/kg every 3 weeks after initiation of 18 mg/kg loading dose combined with continuous daily dose of erlotinib (150 mg QD) until any of the withdrawal criteria are met. Adverse events (AEs) were assessed using CTCAE v4.0 and tumor response was assessed using RECIST v1.1. Full pharmacokinetic sampling and serum biomarker analyses were mainly performed during cycle 1 and 2.

**Results:**

Total of six EGFR-mutant NSCLC patients including one EGFR-TKI naïve patient received patritumab Process 2 formulation combined with erlotinib. No dose-limiting toxicities were observed. The most frequent AEs were gastrointestinal or skin toxicities, which were generally mild and manageable. One patient discontinued from study due to reversible grade 3 interstitial lung disease. The mean area under the curve (AUC) value was 2640 μg/day/mL; the Cmax value was 434 μg/mL, respectively. The median progression-free survival (95% confidence interval) was 220.0 (100.0–363.0) days. HER3 ligand heregulin was detected in serum from only a patient that maintained most durable stable disease.

**Conclusions:**

Patritumab Process 2 formulation in combination with erlotinib was well tolerated compatible with favorable PK profile in Japanese patients with advanced NSCLC.

## Introduction

The human epidermal growth factor receptor-3 (HER3) is a member of the HER (EGFR/ErbB) receptor family consisting of four closely related type 1 transmembrane receptors (EGFR, HER2, HER3, and HER4). HER receptors are part of a complex signaling network intertwined with the Ras/Raf/MAPK, PI3K/AKT, JAK/STAT, and PKC signaling pathways. HER3 is expressed in many normal tissues and in a variety of solid tumors, including non-small cell lung cancer (NSCLC) [1–4], and increased levels of HER3 have been associated with a negative clinical prognosis, including survival in several tumor types [5–8]. HER3 is the only HER family member that lacks tyrosine kinase activity because of an amino acid substitution in the conserved kinase domain. Thus, interactions of HER3 with binding partners are essential for its biological activity [9]. In particular, HER3 potently activates downstream phosphatidylinositol-3-kinase (PI3K) and AKT pathway signaling by directly binding to PI3K through six consensus phosphotyrosinesites [10]. Recent preclinical and clinical data also suggest that HER3 is involved in resistance to other HER receptor-targeted therapeutics [11–15]. Since HER3 has limited kinase activity, several newly developed monoclonal antibodies (mAbs) are being explored to target HER3 for cancer therapy.

Patritumab (U3-1287) is a fully human monoclonal immunoglobulin G1 (IgG1) antibody directed against HER3, thereby inhibiting ligand binding [heregulin alpha (HRG-α) and heregulin beta (HRG-β)] and receptor activation and induces HER3 down-regulation. Functionally, patritumab inhibits tumor cell proliferation, survival, and anchorage-independent growth in vitro, and inhibits growth of HER3 expressing xenograft tumor models in vivo [16–19]. In addition, the combined use of patritumab with erlotinib, an epidermal growth factor receptor-tyrosine kinase inhibitor (*EGFR*-TKI), led to increased inhibition of tumor proliferation, compared with patritumab alone [18].

In a first-in-human phase 1 study (ClinicalTrials.gov Identifier: NCT00730470), the safety and tolerability of patritumab were evaluated up to the dose of 20 mg/kg without dose-limiting toxicities (DLTs) [20]. In another phase 1 study (ClinicalTrials.jp Identifier: JapicCTI-101,262) in Japanese patients, patritumab was well tolerated up to 18 mg/kg without DLTs and PK profile was similar to the US phase I study [21]. Regarding the combined approaches of former formulation of patritumab (patritumab Process 1) with other molecular targeting agents, both erlotinib in patients with NSCLC and trastuzumab and paclitaxel in patients with HER2 overexpressing metastatic breast cancer were evaluated [22, 23]. The drug substance and product for use in nonclinical studies, phase 1 clinical studies, and phase 2 clinical studies during early phases of development of patritumab were manufactured using Manufacturing Method Process 1. Subsequently, a new method, Process 2 was developed to manufacture the drug substance and product for use in a global phase 3 study, with the aim of increasing the yield of the target protein and improving properties of the drug substance and/or product. Based on both safety profile and clinical activities of patritumab Process 1 combined with *EGFR*-TKI, erlotinib in patients with advanced NSCLC, this study evaluated that the safety and pharmacokinetics of patritumab Process 2 in combination with erlotinib and potential biomarkers related to patritumab were also evaluated.

## Materials and methods

### Patient eligibility

This study was conducted based on the Declaration of Helsinki and the Guidelines for the Clinical Evaluation Methods of Anti-Cancer Drugs in Japan (Japanese Ministry of Health, Labour, and Welfare notification, November 1, 2005). The study was approved by the institutional review board of study site.

The main eligibility criteria were as follows: a histologically or cytologically confirmed diagnosis of stage IIIB/IV NSCLC in a patient who had experienced disease progression while on the standard therapy or in a patient intolerant of, or not eligible for the standard therapy (prior *EGFR*-TKIs therapy allowed); a patient age ≥ 20 years; an Eastern Cooperative Oncology Group (ECOG) performance status of 0 or 1; a life expectancy of more than 3 months; and adequate hematologic, hepatic, and renal functions. The exclusion criteria included the administration of chemotherapy, radiotherapy, or biological therapy in the 4 weeks (2 weeks for palliative radiotherapy and kinase inhibitors) prior to enrollment; other active malignancies; history or presence of interstitial lung disease (ILD); history (within 6 months before enrollment) or presence of severe cardiovascular or cerebrovascular disease, pulmonary thrombosis, deep vein thrombosis, or other clinically severe pulmonary disease; any of the following complications, including clinically severe infections requiring systemic administration of an antimicrobial agent, antiviral agent or other agents; presence of chronic diarrhea, inflammatory bowel disease or partial ileus; presence of peptic ulcer; fluid retention requiring treatment; corneal disease; uncontrolled diabetes mellitus; hypertension; psychiatric symptoms; appositive test for hepatitis B virus surface antigen, hepatitis C virus antibody or human immunodeficiency virus antibody; history of a bleeding diathesis; and history of serious hypersensitivity to drugs containing polysorbate 20. All patients provided informed consent, and the study was conducted in accordance with the current Good Clinical Practice standards. This study was registered at ClinicalTrials.jp Identifier:JapicCTI-152,841.

### Study design and evaluation

This study was an open-label, non-randomized, phase 1 study of patritumab Process 2 formulation of fully human anti-HER3 monoclonal antibody in combination with erlotinib in Japanese patients with advanced NSCLC that was conducted at single site in Japan. The primary objective was to evaluate the safety and tolerability of patritumab Process 2 formulation combined with erlotinib in Japanese patients with advanced NSCLC. Secondary objectives were to assess the PK profile, preliminary tumor response, to evaluate incidence of anti-patritumab antibody, and to explore patritumab-related biomarkers. Patritumab Process 2 formulation was administered as a 60-min intravenous (i.v.) infusion at 18 mg/kg for the initial dose and at 9 mg/kg for the second and subsequent doses every 3 weeks in combination with an oral daily dose of erlotinib 150 mg (150 mg QD). Patients received fixed dose of patritumab Process 2 formulation at 9 mg/kg every 3 weeks after initiation of 18 mg/kg loading dose combined with erlotinib at 150 mg QD in single dose cohort until any of the withdrawal criteria are met. Withdrawal criteria are disease progression, unacceptable toxicity, ILD, dosing postponed/discontinued for more than 3 weeks, subject’s request to withdraw from study treatment, and other instances in which the study cannot be continued in the judgment of the investigator. The initial 21 days after the first administration (cycle 1) were regarded as the DLT evaluation period, and six patients were enrolled at single dose cohort. In the DLT assessments, if none/one of the six patients had a DLT that dose was considered to be tolerable. Adverse events (AEs) were graded using the National Cancer Institute Common Terminology Criteria for AEs, version 4.0. A DLT was defined as any of the following events occurring during cycle 1 (the initial 21 days) as related to either patritumab or erlotinib: (1) grade 3 or higher febrile neutropenia, or persistent (more than 7 days) grade 4 neutropenia; (2) grade 4 thrombocytopenia, or grade 3 thrombocytopenia requiring blood transfusion; (3) uncontrollable grade 3 or higher fatigue, anorexia, nausea, vomiting, skin disorder (e.g., skin eruption, urticaria), and diarrhea despite maximal supportive therapy; (4) grade 3 or higher toxicity, with the exception of (1)–(3) as well as pyrexia without neutropenia, transient electrolyte abnormality, and transient lab-oratory abnormality not requiring treatment and without clinical symptoms; and (5) toxicity requiring suspension of erlotinib therapy for more than 7 days during the DLT evaluation period. Tumor response was determined for all patients with measurable and/or non-measurable lesions according to Response Evaluation Criteria in Solid Tumors (RECIST version 1.1). Tumor measurements by CT or MRI were obtained at baseline, every 6 weeks thereafter and Cycle 1 Day 21.

### Pharmacokinetics

Pharmacokinetics were evaluated in all patients received patritumab Process 2 formulation in combination with erlotinib. Blood samples were collected at pre-dose and 1 (end of infusion), 4, 7, 24, and 72 h after the start of first dose infusion, on Days 8 and 15 of Cycle 1, and on Day 1 of Cycles 2, 3, and 4. Serum concentrations of patritumab Process 2 formulation were determined by enzyme-linked immunosorbent assay (ELISA). Pharmacokinetic parameters after the first dose were calculated by non-compartmental analysis using WinNonlin (Ver.6.2 CERTARA G.K., Japan).

Pharmacokinetic statistical analyses were performed using SAS System Release 9.2 (SAS Institute Japan Ltd., Tokyo, Japan).

### Biomarkers

Serum soluble HER3 and the HER3 ligand, heregulin (HRG) levels were also evaluated in all patients. Blood for serum biomarkers was collected on Day 1 (before administration), 8, 15, and 21 of Cycle 1, and Day 21 of Cycle 2 and changes in both soluble HER3 and HRG serum levels were evaluated. Soluble HER3 levels were measured by enzyme-linked immunosorbent assay (ELISA). In addition, soluble HRG was also evaluated immunologically according to the previous report [24, 25].

### Statistical method

All patients who received study medication were included in the analysis of safety and efficacy. Safety and efficacy statistical analyses were performed by SAS System Release 9.2 (SAS Institute Inc., Cary, NC, USA).

## Results

### Patient characteristics

Six Japanese patients with advanced NSCLC were enrolled and were evaluated in this study. The baseline characteristics of the patients are summarized in Table 1. The age range was 54.0–78.0 years (median: 72.5 years), and all six patients harbored *EGFR* mutation (exon 19 deletion,* n* = 2; L858R, * n* = 4; T790M, * n* = 1). Five patients received prior *EGFR*-TKI therapies treated by at least any one of gefitinib, erlotinib, and afatinib, and one patient was an *EGFR*-TKI therapy naïve patient. The median number of prior chemotherapy regimens was 2 (range, 1 − 5). At the time of data cutoff, all patients had discontinued treatment: one because of an adverse event (grade 3 ILD), one because of patient withdrawal and four because of disease progression.

**Table 1:** Baseline patient characteristics

Characteristic	Value
Number of patients	6
Age, year	
Median	72.5
Range	54–78
Sex	
Male	3
Female	3
Weight, kg	
Median	51.9
Range	42.2 –63.8
Histology	
Adenocarcinoma	6
Tumor stage	
III B	0
IV	6
ECOG Performance status	
0	3
1	3
EGFR mutation genotype	
Wild type	0
Exon 19 deletion	2
L858R	4
T790M	1
Number of prior systemic therapies	
Median	2
Range	1–5
Prior EGFR-TKIs	
Any (gefitinib, erlotinib or afatinib)	5
Gefitinib	4
Erlotinib	2
Afatinib	1

*ECOG* Eastern Cooperative Oncology Group, *EGFR* Epidermal Growth Factor Receptor

### Safety and tolerability

The DLTs occurring during cycle 1 were evaluated. No DLTs were observed in all patients. The AEs reported for all treatment cycles are summarized in Table 2.

**Table 2:** Adverse events in more than 20 % of patients

Preferred term	*N* = 6 *n *(%)
Diarrhoea	6 (100.0)
Stomatitis	6 (100.0)
Dermatitis acneiform	6 (100.0)
Dry skin	5 (83.3)
Paronychia	4 (66.7)
Nail infection	2 (33.3)
Tumour pain	2 (33.3)
Decreased appetite	2 (33.3)
Insomnia	2 (33.3)
Malaise	2 (33.3)
Weight decreased	2 (33.3)

Preferred Terms are coded using MedDRA version 19.0

The most common overall AEs (≥ 50 %) were diarrhea, stomatitis, dermatitis acneiform, dry skin, and paronychia which were generally mild and manageable. Most of the AEs were related to both patritumab and erlotinib and were generally mild and manageable. No grade 4 or grade 5 AEs occurred in this study. One patient discontinued both patritumab and erlotinib treatment because of a drug-related AE. An elderly female patient with gefitinib-pretreated advanced NSCLC developed grade 3 ILD (onset, day 86); after the discontinuation of both patritumab and erlotinib treatment, steroid administration was conducted and ILD was resolving. No patients developed anti-patritumab antibodies after the administration of patritumab Process 2 formulation in this study.

### Pharmacokinetics

**Fig. 1:**
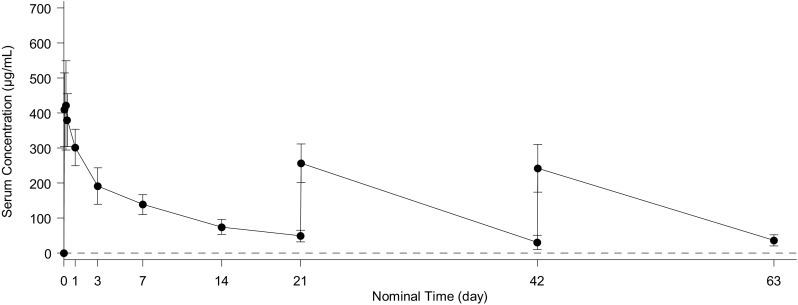
The mean ± sd serum concentration for patritumab Process 2 formulation versus time

Mean +/− sd serum concentration for patritumab Process 2 formulation versus time is shown in Fig. 1, and descriptive statistics for the PK parameters are summarized in Table 3. The mean area under the curve (AUC) value was 2640 µg day/mL; the C_max_ value was 434 µg/mL; and the terminal half-live was 9.18 days, respectively.

**Table 3:** Summary of pharmacokinetic parameters of patritumab (U3-1287) Process 2 formulation in Japanese patients with advanced NSCLC

	C_max,_ µg/mL, mean (CV%)	T_max,_ days, mean (range)	AUC_0 − 21day_, µg·day/mL, mean (CV%)	AUC_0−∞_, µg·day/mL, mean (CV%)	t_1/2,_ days, mean (CV%)	Vss, mL/kg, mean (CV%)	CL, mL/day/kg, mean (CV%)
(*n* = 6)	434 (27.9)	0.09 (0.05–0.18)	2640 (20.6)	3310 (24.8)	9.18 (23.2)	69.5 (18.1)	5.70 (22.4)

*C*_*max*_ maximum observed serum concentration, *T*_*max*_ time of maximum observed serum concentration, *AUC*_*0* − *21day*_ area under the concentration–time curve from day 0 to day 21, *AUC*_*0*−∞_ area under the concentration–time curve from day 0 to infinity, *t*_*1*/*2*_ elimination half-life, *Vss* the terminal phase volume, *CL* clearance, *CV* coefficient of variation

### Efficacy

One partial response (PR) and five cases with stable disease (SD) were observed. The PR was observed in an *EGFR*-TKI naïve patient who had a tumor with an *EGFR*-activating mutation (L858R). Among the five SD patients, all patients received prior *EGFR*-TKIs (treated by at least any one of gefitinib, afatinib, and erlotinib) treatment (exon 19 deletion,* n* = 2; L858R,* n*  = 3, T790M,* n* = 1). The median progression-free survival (PFS) (95 % confidence interval) was 222.0 (100.0–363.0) days.

### Biomarker analysis (serum HER3 and heregulin)

Soluble serum HRG level was detectable in only a patient, subject No. 2, prior the administration (5,190.1 pg/ml), although was not detectable in other samples from five patients (Table 4). In addition, this patient maintained a most durable stable disease for 363 days among six patients, although tumor already acquired a resistance to gefitinib. The soluble HRG level was maintained on during Cycles 1 and 2 in a patient subject No. 2 (7411.2 pg/ml at Day 8 of Cycle 1; 6870.5 pg/ml at Day 15 of Cycle 1; 5399.1 pg/ml at Day 21 of Cycle 1; and 5329.5 pg/ml at Day 21 of Cycle 2). Soluble HER3 level was various in serum obtained from six patients prior the administration (mean 7,475.8 pg/ml; 3,164.0–25,005.5 pg/ml, Table 4). Soluble HER3 level increased post the administration (mean 15,585.8 pg/ml at Day 8 of Cycle 1; 18,016.7 pg/ml at Day 15 of Cycle 1; 18,100.1 pg/ml at Day 21 of Cycle 1, Supple. Table 2).

**Table 4:** Patient characteristics and soluble HER3 expression in serum

Subject no.	EGFR genotype	Prior EGFR-TKI	Best overall response	Progression-free survival (days)	HER3 concentration in serum, pg/ml				
					Day 1 of Cycle 1	Day 8 of Cycle 1	Day 15 of Cycle 1	Day 21 of Cycle 1	Day 21 of Cycle 2
1	L858R	Naïve	PR	187	4493	13,476	15,741	17,527	19,060
2	Exon 19 deletion	Treated	SD	363	3862	12,058	16,284	16,366	18,687
3	L858R	Treated	SD	100	4023	11,439	14,411	14,464	15,580
4	L858R	Treated	SD	297	3164	9002	11,587	12,914	13,399
5	L858R/T790M	Treated	SD	257	25,006	35,006	37,062	33,052	35,890
6	Exon 19 deletion	Treated	SD	101	4308	12,534	13,016	14,280	18,544

*PR* partial response, *SD* stable disease

## Discussion

This phase 1 study was conducted primarily to evaluate the safety and tolerability of patritumab Process 2 formulation combined with EGFR-TKI, erlotinib in Japanese patients with advanced NSCLC. The safety and tolerability, PK, anti-patritumab antibody, tumor response, and biomarkers, including sHER3 and sHRG, were explored in this study. Patritumab Process 2 formulation was developed to manufacture the drug substance with the aim of increasing the yield of the target protein and improving properties of the drug substance and/or product.

Regarding safety and tolerability, no DLTs were reported at all patients (patritumab Process 2 formulation at 9 mg/kg every 3 weeks after initiation of 18 mg/kg loading dose with oral daily dose of erlotinib 150 mg). The most common AEs in this study were gastrointestinal and skin toxicities, which were generally mild and manageable and most AEs in this study were similar to the well-known side effects of *EGFR*-TKIs. No treatment-related deaths due to AEs were reported. Some SAEs were reported, including grade 3 ILD, which was related to either patritumab or erlotinib treatment. An elder female patient with gefitinib-pretreated advanced NSCLC developed grade 3 ILD (onset, day 86); after the discontinuation of both patritumab and erlotinib treatment, steroid administration was conducted and ILD was resolving.

PK parameters were calculated by non-compartmental model-based analysis, and AUC_0−21day_ and C_max_ were compared with existing data of former Process 1 formulation combined with erlotinib in Japanese patients with non-small cell lung cancer. The AUC_0−21day_ and Cmax (Mean ± SD) of the Process 2 formulation were 2640 ± 544 µg day/mL and 434 ± 121 µg/mL, respectively, suggesting almost comparable exposure obtained with Process1 formulation (AUC_0−21day_ and C_max_: 2480 ± 420 µg day/mL and 400 ± 46.7 µg/mL, respectively) [22]. Furthermore, no neutralizing antibodies were detected in patients in this study after patritumab Process 2 formulation administration, as assessed by an anti-patritumab antibody and cell-based bioassay, similar to findings in the previous studies.

In regard to the clinical efficacy of the combined treatment, 1 PR and 5 cases with SD were observed among six patients. The PR patient had a tumor with an *EGFR*-activating mutation (L858R) and *EGFR*-TKI naïve setting. Among six patients, five patients had available information about T790M status of their tumor by conducting pre- or post-biopsy of tumor tissue when patients developed disease progression against preceding *EGFR*-TKIs therapy. Although this was limited patients’ number, one key remarkable point about potential clinical efficacy in this study is that durable progression-free survival was observed (363, 100, and 297 days) in three T790M wild-type *EGFR*-TKI refractory patients. These results were encouraging, because they were similar or superior to those obtained with use of the third generation *EGFR*-TKI, osimertinib (AZD9291) in recent clinical study in patients with previously treated NSCLC and sub-population analysis of patients with no detectable *EGFR* T790M (69 % of the patients had an estimated response duration of 6 months or longer, with a median progression-free survival of 2.8 months (95 % CI, 2.1 to 4.3; 71 % maturity) in 62 patients with no detectable *EGFR* T790M [26].

The current study observed that a patient had a high level of soluble HRG in serum and maintained most durable stable disease, although tumor already acquired a resistance to gefitinib. Previous studies proved that HRG expression level correlated with the efficacy of several kinds of anti-HER3 antibody preclinically as well as clinically [25, 27–30]. U3-1287 also inhibited cell-proliferation in aberrantly heregulin-expressing NSCLC cells [28]. Furthermore, Patritumab combination with erlotinib demonstrated a significantly improved progression-free survival in patients with advanced NSCLC and high level of soluble HRG in serum compared to placebo combination [25]. Although the current study analyzed limited samples, patritumab plus erlotinib might be optimal in patients with *EGFR*-mutant NSCLC and high level of HRG expression.

In conclusion, patritumab Process 2 formulation at a dose of 18 mg/kg for the initial dose and at 9 mg/kg for the second and subsequent doses every 3 weeks in combination with an oral daily dose of erlotinib 150 mg was determined to be feasible in regard to the tolerability in Japanese patients with advanced NSCLC. Although some limitations including small patients number exist in this study, preliminary demonstration of both favorable PK profiles and the efficacy of the combined treatment was encouraging, potentially in NSCLC patients with *EGFR*-activating mutations, where prior *EGFR*-TKI treatment failed regardless of *EGFR* T790M status.
